# Transcriptomic Profiling Identifies Neutrophil-Specific Upregulation of Cystatin F as a Marker of Acute Inflammation in Humans

**DOI:** 10.3389/fimmu.2021.634119

**Published:** 2021-04-01

**Authors:** Andrew J. Sawyer, Mathieu Garand, Damien Chaussabel, Carl G. Feng

**Affiliations:** ^1^ Immunology and Host Defense Group, Discipline of Infectious Diseases and Immunology, Faculty of Medicine and Health, The University of Sydney, Sydney, NSW, Australia; ^2^ Tuberculosis Research Program, Centenary Institute, The University of Sydney, Sydney, NSW, Australia; ^3^ Immunology Program, Sidra Medicine, Doha, Qatar

**Keywords:** neutrophil, transcriptome, cystatin F, data mining, meta-analysis, literature analysis

## Abstract

Cystatin F encoded by *CST7* is a cysteine peptidase inhibitor known to be expressed in natural killer (NK) and CD8^+^ T cells during steady-state conditions. However, little is known about its expression during inflammatory disease states in humans. We have developed an analytic approach capable of not only identifying previously poorly characterized disease-associated genes but also defining regulatory mechanisms controlling their expression. By exploring multiple cohorts of public transcriptome data comprising 43 individual datasets, we showed that *CST7* is upregulated in the blood during a diverse set of infectious and non-infectious inflammatory conditions. Interestingly, this upregulation of *CST7* was neutrophil-specific, as its expression was unchanged in NK and CD8^+^ T cells during sepsis. Further analysis demonstrated that known microbial products or cytokines commonly associated with inflammation failed to increase *CST7* expression, suggesting that its expression in neutrophils is induced by an endogenous serum factor commonly present in human inflammatory conditions. Overall, through the identification of *CST7* upregulation as a marker of acute inflammation in humans, our study demonstrates the value of publicly available transcriptome data in knowledge generation and potential biomarker discovery.

## Introduction

Transcriptomic profiling has been widely used over the past two decades to uncover the expression and function of genes across the genome. Each transcriptomic profiling study generates enormous amounts of data, the bulk of which has been published in online repositories and is available for download free of charge. The NCBI Gene Expression Omnibus (GEO), for example, is an online database containing more than 58,000 series datasets from human studies, comprising 2 million individual samples. These include datasets from across the spectrum of human diseases, tissue types and cell types. Transcriptomic data generated from cells following stimulation or from gene-targeted cells provide additional insights into gene regulation. While transcriptomic datasets have typically been interrogated by the original researchers to identify pathways/genes of interest, most of the data remains incompletely explored and utilized. This wealth of data remains available online and represents a valuable resource for life scientists exploring a gene or pathway of interest. For example, investigations can be used to generate informed hypotheses that guide future studies.

Of particular interest are genes that are found to be differentially expressed despite no prior association with the conditions under investigation. In this case, public transcriptomic datasets can be used to validate findings, facilitate further clarification of the specificity of gene expression across cell types and diseases, and even provide insights into gene regulation. Some studies have already successfully identified a gene of interest across a small cohort of GEO datasets and been used to formulate a hypothesis for gene function (1, 2).

Here, we demonstrate an analytic pipeline that allows identification of differentially expressed genes that are yet to be characterized, then define mechanisms regulating their expression across a cohort of 43 publicly available transcriptomic datasets. We discovered that the expression of *CST7* in whole blood was markedly upregulated in sepsis as well as across a range of bacterial, viral, and sterile inflammatory conditions. Cystatin F, encoded by *CST7* is a cathepsin C-directed cysteine protease known to regulate cytotoxicity in natural killer (NK) cells (3, 4). While cathepsin C is involved in serine peptidase activation in NK cells and neutrophils, the significance and regulation of cystatin F expression in neutrophils remains unexplored. Our analysis revealed that this *CST7* upregulation is neutrophil-specific and regulated by host factors. In this study, we demonstrate that transcriptomic profiling using publicly available datasets is a robust approach to identify associations of unknown genes with human diseases.

## Methods

### Literature Search

PubMed was searched to retrieve the literature associated with each gene using a query that consisted of a concatenation of its official name, symbol and aliases retrieved from NCBI gene. Using *CST7* as an example, the following query was applied: CST7 [tw] OR ‘cystatin F’ [tw] OR Leukocystatin [tw]. The text word [tw] function was used to restrict the search to the title and abstract.

### Single-Cohort Transcriptomic Analysis

All datasets used in this study were collected from the NCBI GEO (**Supplementary Table 1**). All single-cohort analysis was performed in R and datasets were downloaded using the GEOquerey (5) package. Normalized expression counts of genes of interest were exported for plotting. Where necessary, datasets were log-transformed and DEGs were identified using the Limma package (6) and P values were adjusted with a Benjamini Hochberg false discovery rate of 0.01.

### Multicohort Transcriptomic Analysis

All Multicohort analyses were performed in R using the Metaintegrator package (7). Datasets were accessed from GEO using the “getGEOData” function and samples were separated into control and non-control groups using the “classFunction” function. Meta-analysis was performed using the “RunMetaAnalysis” function and forest plots were previewed with the “forestPlot” function. A summary effect size for genes was determined based on the standardized mean difference in each cohort. Effect sizes for DEGs between septic patients and controls across the nine whole blood sepsis cohorts were identified using the “filterGenes” function with an effect size threshold of 1 and false discovery rate threshold of 0.05. Heatmaps of sepsis DEG expression across other disease states were generated using the heatmapPlot function with “diplayPooled = FALSE” to maintain the original gene order.

### Software

Transcriptomic analysis was performed in R 4.0.2. For single-cohort group comparisons, *t*-tests were performed using GraphPad Prism 7.02. All plots were generated using GraphPad Prism 7.02. Figures were prepared using Microsoft PowerPoint 2019.

## Results

### Cystatin F Is Upregulated in Neutrophils Following Stimulation With Septic Plasma and in Circulating Leukocytes of Sepsis Patients

**Figure 1:**
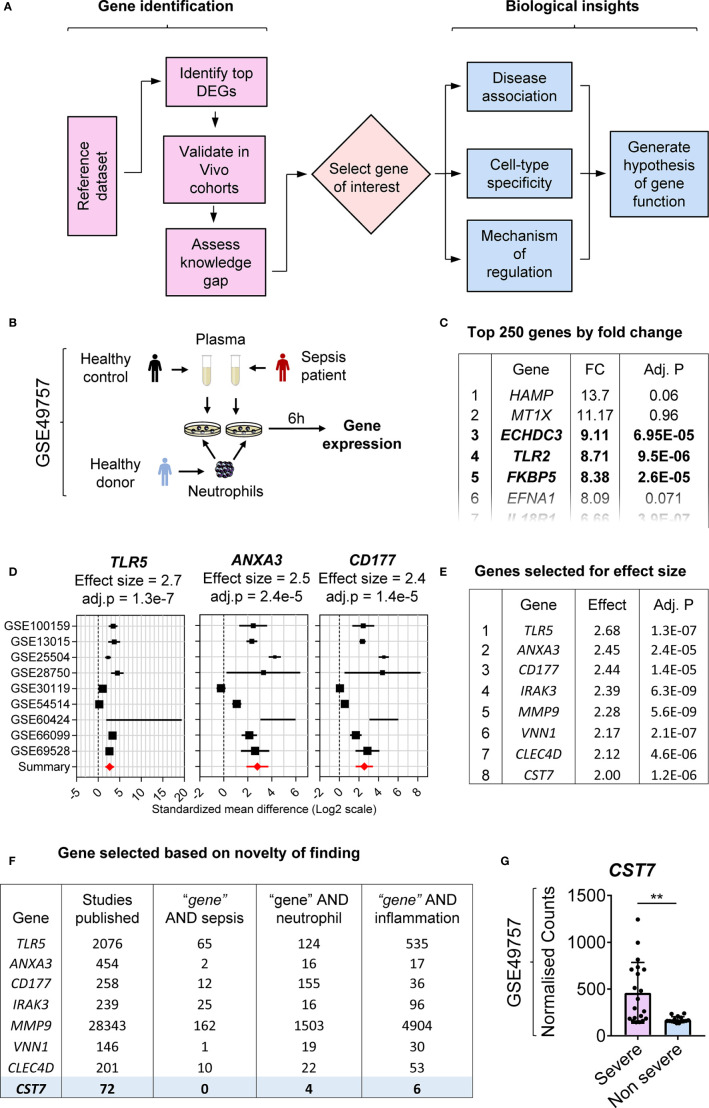
Identification of *CST7* as a previously unknown neutrophil-specific, sepsis-associated gene. **(A)** Flow chart of the process employed to identify a gene of interest and assess its regulation. **(B)** Schematic overview of the experimental design for the reference dataset (GSE49757) used in this study. Purified neutrophils from healthy adult donors were stimulated with plasma from either septic or healthy individuals for 6 h and gene expression was measured by microarray. **(C)** Genes were sorted according to fold change in expression and the top 250 genes with a significant change in expression were selected for further assessment. The level of statistical significance was determined using Student’s *t*-test and *P* < 0.05 was considered to indicate statistical significance. **(D)** Gene expression profiles across nine transcriptomic datasets comparing whole blood samples from patients with sepsis to healthy controls are represented in forest plots. Cohort identifiers are shown on the Y-axis and closed black squares represent the standardized mean difference. Square size is proportional to the sample size and whiskers represent a 95% confidence interval. The summary (closed red diamond) represents the overall change in gene expression across cohorts and is also shown as the effect size above each plot along with its corresponding adj. *P*-value. **(E)** The top 250 genes identified in panel B were sorted according to overall effect size across sepsis cohorts. **(F)** For each gene, PubMed was searched to identify both the total number of articles and the number of articles linking the gene with each of the terms “sepsis”, “neutrophil” and “inflammation” in the title and abstract. Genes were searched in order from the highest effect size until a gene with adequate novelty (defined by the number of publications) was identified. **(G)** *CST7* expression was compared between patients with severe sepsis vs. non-severe sepsis in the reference dataset (GSE49757). Each symbol represents an individual donor, while bars represent the mean ± standard deviation. The level of statistical significance was determined using Student’s *t*-test and *P* < 0.05 was considered to indicate statistical significance. **P < 0.01.

We have shown previously that transcriptomic changes in public datasets can be explored for biomarker discovery by identifying previously uncharacterized genes (1, 2). Here we demonstrate a two-staged approach of literature-searches and public-data mining, which enables not only to identify a novel gene of interest but also to define regulatory mechanisms controlling its expression (**Figure 1A**). To identify an initial cohort of genes upregulated during inflammation, a dataset deposited in the NCBI GEO database (GSE49757) was chosen. Neutrophils from healthy donors were stimulated with plasma from either healthy controls or bacterial culture-confirmed sepsis patients and mRNA expression was measured using microarray (**Figure 1B**). Genes were sorted according to the fold change in expression and the top 250 genes were selected for further analysis (**Figure 1C**).

To extend the *in vitro* observations and identify potential candidate genes for further study, validation was performed in publicly available datasets generated using whole blood samples from healthy controls and sepsis patients. Nine datasets were selected from the NCBI GEO and multicohort analysis was performed to generate a mean fold change in expression across all cohorts. The top 250 genes were ranked according to fold change in expression in this cohort (**Figure 1D**). The highest ranked genes in terms of the effect size were considered as the strongest candidates for further investigation (**Figure 1E**). The final step in the process of selecting a gene of interest was to assess the current knowledge on each identified gene. PubMed was searched to identify the total number of articles relating to each gene and alternate names for each gene were identified through NCBI Gene and added to the search. Genes for which a very small number of articles were identified (<10) were excluded as it was considered unlikely that sufficient information about these gene would be available to inform future studies. Additional searches were carried out for each gene paired with the search terms “sepsis”, “neutrophil” and “inflammation” to identify previous gene associations. The number of articles was counted, and a score applied to each gene based on the number of related articles. Genes were examined in order starting from the gene with the highest fold change in expression and those with known associations to sepsis or neutrophils were excluded (**Figure 1F**). *CST7* (Cystatin F) was ranked 8^th^ in terms of fold change in expression and 72 articles were identified that referred to the gene. Among these, six publications were related to inflammation; however, none of these articles contained the term “sepsis” or linked cystatin F directly to the term neutrophil. *CST7* was therefore deemed the most eligible for further investigation. We further confirmed that *CST7* expression was significantly increased in neutrophils following stimulation of plasma from patients with severe sepsis compared with those with non-severe sepsis (**Figure 1G**).

### The Significance and Regulation of Cystatin F Induction Are Poorly Defined

**Figure 2:**
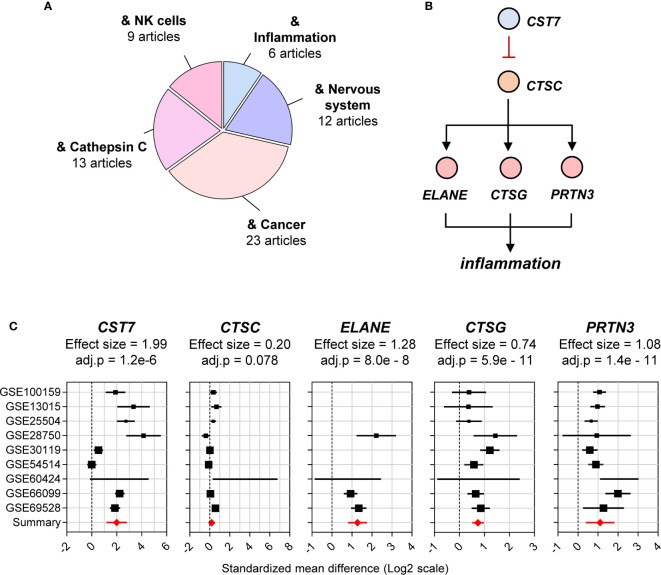
Cystatin F is identified as a cathepsin C-directed serine protease inhibitor. **(A)** The total number of articles on *CST7* (72 articles) was curated and major reported biological functions were summarized. The size of each segment in the pie chart corresponds to the number of articles matching *CST7* in relation to the indicated area. **(B)** The proposed function of the *CST7* protein indicating its ability to inhibit *CTSC* protein. *CTSC*, which is known to activate the neutrophil serine proteases *ELANE*, *PRTN3* and *CTSG*, can increase inflammation. **(C)** Forest plots of *CST7*, *CTSC*, *ELANE*, *PRTN3* and *CTSG* gene expression across sepsis cohorts. The plots were constructed as described in **Figure 1D**.

To inform further exploration of public data, a profile of the current knowledge of *CST7* was constructed. The 72 articles that referred to *CST7* were examined for major research themes and tallied (**Figure 2A**). The cystatins consist of three families of proteins that bind reversibly with high-affinity to inhibit multiple cathepsin family members (8) Cystatin F is unique among the cystatins in that its expression is limited to leukocytes (9) and because it is secreted in an inactive form that can be internalized and subsequently activated in target cells (10, 11). In the context of malignancy, increased levels of *CST7* have been correlated with a poorer prognosis in liver metastasis following colorectal cancer (12) and with improved survival in the context of pancreatic ductal adenocarcinoma (13) and hepatocellular carinoma (14). Additionally, in the context of neuroinflammation, *CST7* is upregulated during CNS demyelination and is associated with areas of remyelination (15, 16). While *CST7* has not been studied in the context of other inflammatory conditions, it has been identified as playing a role in granulocyte inflammation, specifically as an inhibitor of cathepsin C (17).

Cathepsins consist of a large family of cysteine proteases and play a diverse set of roles in both physiological and pathophysiological conditions. In the immune system, they are involved in antigen processing and presentation, activation of pattern recognition receptors and as cytotoxic devices utilized by both the host and pathogens (18). Cathepsin C is a component of the granules in cytotoxic leukocytes and functions as a key activator of other inflammatory granules in these cells (17). Cystatin F can inhibit cathepsin C in NK cells, thereby inducing an impaired cytotoxic state known as split anergy (3, 19). High levels of cystatin F in CD8^+^ T cells are also correlated with decreased cytotoxicity (20).

In neutrophils, cathepsin C is required for activation of neutrophil serine proteases including Elastase, neutrophil expressed (*ELANE*), Cathepsin G (*CTSG*) and Proteinase 3 (*PRTN3*) in the inflammatory granules (17, 21, 22). However, whether cystatin F inhibits cathepsin C in neutrophils or exerts anti-inflammatory effects in these cells remains unknown (**Figure 2B**). Due to their potential connection with *CST7*, we also examined *CTSC*, *ELANE*, *CTSG* and *PRTN3* in the public data cohorts in the remainder of this study. Based on the previous cohort of sepsis datasets, *CST7* was significantly upregulated (effect size = 1.99) in patients with sepsis compared with healthy controls, while there was no significant difference in *CTSC* expression between the two groups. *ELANE*, *CTSG* and *PRTN3* were significantly upregulated (effect size = 1.28, 0.74 and 1.08, respectively) (**Figure 2C**).

### Cystatin F Is Consistently Upregulated Across Bacterial, Viral, and Sterile Systemic Inflammation

**Figure 3:**
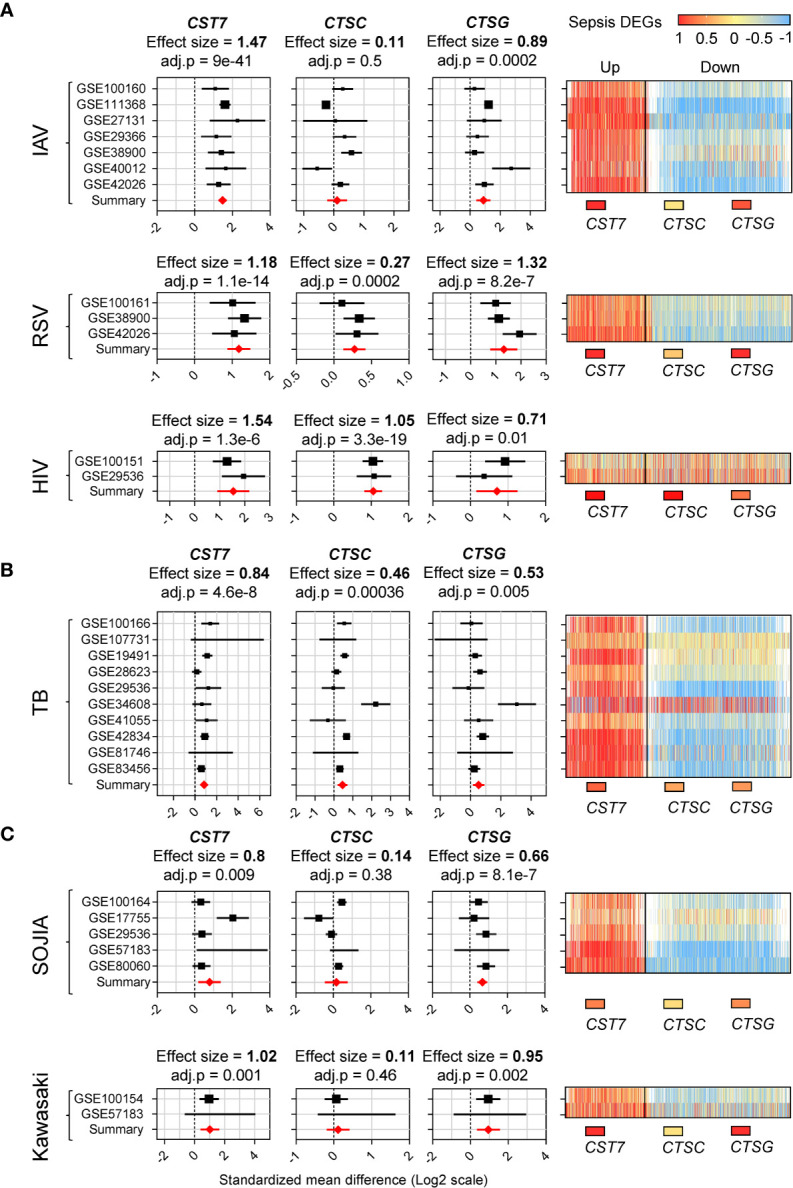
*CST7* is upregulated across a spectrum of acute inflammatory diseases. **(A)** Gene expression profiles for *CST7*, *CTSC*, and *CTSG* across 10 transcriptomic datasets comparing whole blood samples from patients with TB to healthy controls are represented in forest plots constructed as described in **Figure 1D**. An accompanying heatmap represents the expression of the 2,140 DEGs across the sepsis cohorts (X-axis, separated into up/downregulated expression during sepsis) in each TB cohort (Y-axis). This was repeated in **(B)** with transcriptomic datasets comparing in whole blood samples from IAV patients to healthy controls, RSV patients to healthy controls and HIV patients to healthy controls. This was also repeated in **(C)** with transcriptomic datasets comparing whole blood samples from SOJIA patients to healthy controls and Kawasaki disease patients to healthy controls.

Having identified *CST7* as an upregulated gene in whole blood during sepsis, we sought to determine whether the change was restricted to sepsis. We selected six inflammatory diseases for further examination, each of which was represented by at least two cohorts on the GEO. The diseases were categorized as bacterial, viral or sterile inflammation. All the selected cohorts compared whole blood samples from patients and healthy controls. The 2,140 genes that were found to be differentially expressed in the sepsis cohort were measured across each of the other disease cohorts. Heat maps of the similarity between the gene expression profile of each disease state and sepsis are presented in **Figure 3**.

*CST7* expression was significantly increased in all conditions, with the highest effect size during viral-induced inflammation (**Figure 3A**). *CST7* was increased during infection with influenza A virus (IAV), respiratory syntactical virus (RSV) and human immunodeficiency virus (HIV) infection (effect size = 1.47, 1.18, 1.54 respectively). Interestingly, *CTSC* expression did not change during IAV infection, but was significantly increased during RSV and HIV infection. *PRTN3*, *ELANE* and *CSTG* were significantly increased in all three viral infection cohorts. In tuberculosis (TB), *CST7* was significantly upregulated (effect size = 0.84). In contrast, there were no changes in *CTSC* or *CTSG* expression, although expression of *PRTN3* and *ELANE* were increased (**Figure 3B**). Finally, in the case of sterile inflammation, *CST7* expression was increased in both systemic onset idiopathic juvenile arthritis (SOJIA) and Kawasaki disease (effect size = 0.6 and 1.02 respectively). *CTSC* was not changed in either cohort, while CTSG and *PRTN3* were significantly upregulated in both cohorts and *ELANE* was significantly upregulated during SOJIA (**Figure 3C**).

### Cystatin F Upregulation Is Neutrophil-Specific

**Figure 4:**
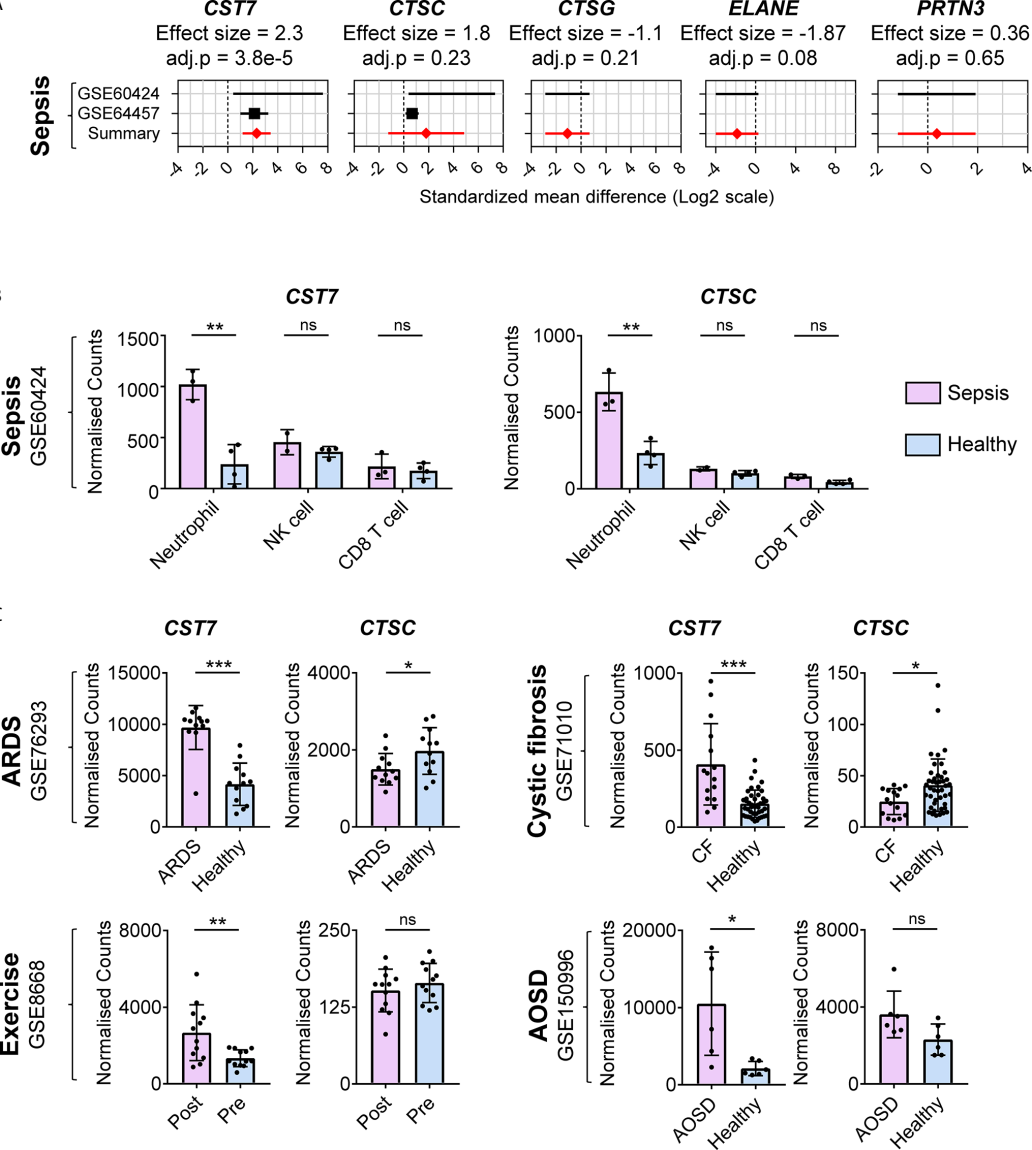
*CST7* upregulation during inflammation is neutrophil-specific. **(A)** Gene expression profiles for *CST7*, *CTSC*, *CTSG*, *ELANE*, and *PRTN3* across two datasets comparing purified neutrophil populations from patients with sepsis and healthy controls are represented in forest plots constructed as described in **Figure 1D**. **(B)** Normalized expression counts of *CST7* and *CTSC* in purified neutrophils, NK cells and CD8^+^ T from healthy controls compared to those from patients with sepsis. **(C)** Expression of *CST7* and *CTSC* in purified neutrophils comparing patients with ARDS (GSE76293), CF (GSE71010) and SOJIA (GSE103170) to healthy controls as well as comparing healthy individuals pre- and post-exercise (GSE8668). In panel B and C each symbol represents an individual donor, while bars represent the mean ± standard deviation. The level of statistical significance is indicated on the plot: *P < 0.05 **P < 0.01 ***P < 0.001, ns, not significant.

Having established that *CST7* is upregulated in bulk leukocyte preparations during inflammation, we next determined whether this change is restricted to certain leukocyte populations in the blood. Given the initial finding that septic plasma induced upregulation of *CST7* in neutrophils, we identified datasets comparing purified neutrophils from healthy controls and sepsis patients. Two datasets were identified and *CST7* was found to be significantly upregulated in neutrophils from sepsis patients compared to those from healthy controls (effect size = 2.3). However, there were no changes in the expression levels of *CTSC*, *CTSG*, *PRTN3* and *ELANE* in neutrophils during sepsis compared to those from healthy controls (**Figure 4A**).

We next examined whether expression of *CST7* is modulated in other leukocyte populations during sepsis using one cohort from the previous analysis that also included purified CD8^+^ T cells, NK cells, CD4^+^ T cell and monocytes as well as neutrophils. While NK cells expressed the highest *CST7* levels under healthy conditions, there was a significant increase in *CST7* expression in neutrophils during sepsis and these cells became the predominant contributor of *CST7* expression (**Figure 4B**). There was no change in *CST7* expression in NK cells or CD8^+^ T cells during sepsis. *CST7* was expressed lowly in in CD4^+^ T cells and monocytes and its expression in these cells did not change during sepsis (Data not shown). Given this neutrophil-specific increase during sepsis, we next sought to determine whether *CST7* levels were increased in neutrophils under other inflammatory conditions. We examined four cohorts comparing gene expression from purified neutrophils during acute respiratory distress syndrome (ARDS), adult-onset Still’s disease (AOSD) and cystic fibrosis (CF) compared to purified neutrophils from healthy controls as well pre- and post-exercise. In all these conditions, *CST7* expression was significantly increased, suggesting neutrophil-specific upregulation of *CST7* is positively associated with inflammatory conditions (**Figure 4C**).

### Cystatin F Expression During Inflammation Is Regulated by Host Factor(s)

**Figure 5:**
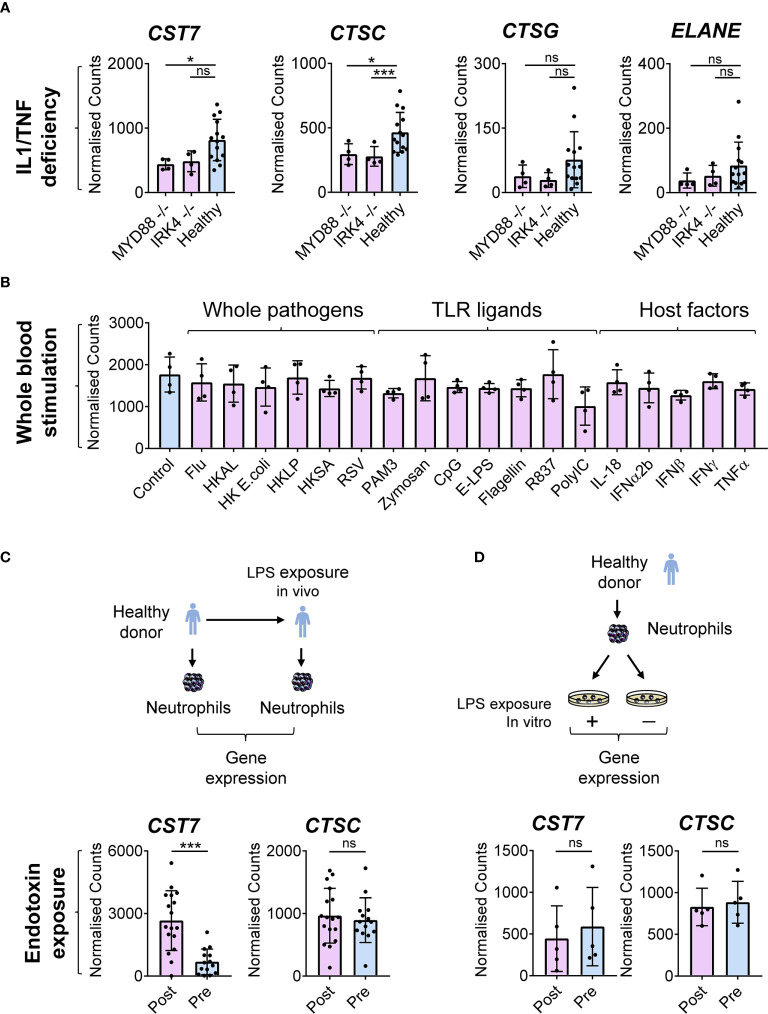
*CST7* upregulation during inflammation is regulated by host factors **(A)** Baseline gene expression of *CST7*, *CTSC*, *CTSG* and *ELANE* in whole blood samples form a cohort of healthy controls or patients with either complete MYD88 deficiency or complete IRAK-4 deficiency. **(B)** *CST7* expression in whole blood cells following stimulation *in vitro* with heat-killed (HK) whole pathogens, purified TLR ligands and host cytokines (GSE30101). Whole blood from four healthy donors was incubated for 6 h with one of the listed stimuli before gene expression was measured by microarray. **(C)** Expression of *CST7* and *CTSC* in a circulating neutrophils from healthy individuals taken prior to or 16 h after bronchoscopic instillation of endotoxin (LPS). **(D)** Expression of *CST7* and *CTSC* in purified neutrophils isolated from healthy donors following stimulation with endotoxin *in vitro*. **(A–D)**, each symbol represents an individual donor, while bars represent the mean ± standard deviation. The level of statistical significance is indicated on the plot: *P < 0.05 ***P < 0.001, ns, not significant.

Having identified upregulation of *CST7* during inflammation, we next examined whether public transcriptomic data can be used to shed light on the regulatory mechanisms underpinning this increase in expression. Given that *CST7* is increased under a diverse range of inflammatory conditions, we hypothesized that a broadly acting inflammatory mediator may regulate *CST7* expression changes. Indeed, examination of whole blood transcriptomic data from one study showed that defective Toll-like receptor (TLR) and IL-1 signaling due to MYD88 or IRAK deficiency is associated with decreased levels of *CST7* under baseline conditions (**Figure 5A**), suggesting a role for an innate signaling pathway in *CST7* upregulation. Therefore, we analyzed another dataset in which whole blood cells were stimulated with a range of TLR ligands, heat killed pathogens and major pro-inflammatory cytokines. However, none of these factors stimulated any significant change in *CST7* expression (**Figure 5B**). Finally, we analyzed a dataset in which neutrophil gene expression was compared following endotoxin exposure *in vivo* and *in vitro*. We found that when neutrophils were purified from the blood of healthy donors and exposed to endotoxin *in vitro*, no change in *CST7* expression was observed (**Figure 5C**), confirming that microbial products do not contribute to *CST7* induction directly. However, when the donors were exposed to endotoxin by bronchoscopic instillation *in vivo* and circulating neutrophils were purified from the blood and examined 16 h later, *CST7* was significantly upregulated (**Figure 5D**). Together, these findings suggest that an endogenous rather than exogenous factor contributes to the *CST7* upregulation during inflammation.

## Discussion

Cystatin F has been poorly characterized as a protease inhibitor and has not been linked to neutrophils until now. By using public transcriptome data, we discovered that *CST7* expression is robustly increased in neutrophils cultured *in vitro* in the presence of plasma from sepsis patients and also in the circulating neutrophils of sepsis patients *in vivo*. Further analysis using additional datasets established that this neutrophil-specific *CST7* upregulation occurs across a diverse range of sterile and pathogen-driven inflammatory conditions. Publicly available transcriptomic data also showed that this induction is independent of a broad range of microbial products, suggesting that *CST7* upregulation during inflammation is regulated by a circulating endogenous soluble factor. These novel findings suggest that *CST7* is a previously unrecognized major inflammation-responsive gene in humans. Given the known anti-cytotoxic actions of cystatin F in NK cells (3), further in-depth investigations focusing on its expression patterns and functions may reveal fundamental mechanisms regulating inflammation in human disease.

Our literature search of the PubMed database revealed that no link has been established between *CST7* expression in the context of sepsis or in neutrophils. This approach, which has been used in previous studies (1, 2), is a simple and rapid method for establishing novel gene expression in the context of a condition or cell of interest. Subsequent full text searches using Google Scholar revealed that *CST7* was identified as a DEG during sepsis in three studies (23–25). However, in each of these studies *CST7* appeared among dozens of other DEGs and none of these studies commented on the biological relevance of *CST7* upregulation during sepsis. Thus, we consider that *CST7* upregulation in neutrophils is a novel biological finding in the context of sepsis.

Throughout this study we performed multicohort analyses of genes of interest to determine an overall fold change in the expression of a gene across any number of compatible datasets. This type of multicohort gene expression analysis has been used to validate biomarker gene signatures across inflammatory diseases (26, 27), including sepsis (28). Here we apply this approach to a single gene across a variety of different diseases and conditions. Comprehensive exploration of the NCBI GEO was performed to identify biologically relevant datasets for each disease and cell-type explored in this study, which led to the discovery of the novel association between neutrophil-expressed *CST7* and acute inflammation in humans. Using our analysis pathway, we additionally identified novel neutrophil-specific changes in the expression of two other genes, *SAMSN1* and *BATF* during inflammation, and each of these genes could form the basis of an additional study. Overall, the multicohort analysis process is straightforward and provides a wealth of information on gene regulation to inform hypotheses for future experiments. Moreover, the entire process is free and uses publicly available data and software.

With the current knowledge of *CST7* in the literature, the new findings generated from this study can be used to inform hypotheses for future research. Of particular interest, was the ability of cystatin F to inhibit cathepsin C and thus, inhibit the activation of serine proteases (17). While cystatin F can function in an autocrine manner, it can also act in trans, being secreted and taken up by other cells. If the upregulation of *CST7* in neutrophils corresponds to an increase in cystatin F protein, it can be speculated that it acts on the neutrophils themselves, other cells, or both. If neutrophilic cystatin F acts in an autocrine manner, this would be of great interest given its known function to suppress cathepsin C.

We hypothesize that cystatin F suppression of cathepsin C in neutrophils may be an as-yet unknown anti-inflammatory function in neutrophils. If neutrophilic cystatin F is secreted, then it may act to suppress cathepsin C in other cells. Furthermore, if cystatin F is secreted by neutrophils in a tumor microenvironment (14), then it may contribute to pro-tumor immunosuppression. We identified a dataset (GSE101584) for tumor-associated neutrophils in a mouse cancer model (29) and found a significant increase in *CST7* expression compared to that in naïve neutrophils. Given that cystatin F also inhibits cathepsin C in mice (30), this supports the hypothesis that cystatin F plays a role in the tumor microenvironment. The ability of cystatin F to inhibit cathepsin C in neutrophils is a key area for future investigation. To test these hypotheses, future bench-based research is required to determine first whether the increase in *CST7* in neutrophils corresponds to an increase in cystatin F protein and whether the protein is secreted.

In this study we have both identified *CST7* as a gene of interest and assessed its regulation across a variety of public transcriptomic datasets. To date, no role for *CST7* has been established in neutrophils or in any inflammatory disease. Having identified *CST7* upregulation across inflammatory diseases and in a neutrophil-specific manner, we were able to formulate hypotheses about the potential inhibitory activity of cystatin F. Our findings suggest that cystatin F or *CST7* may potentially be used, either independently or in conjunction with C-reactive protein, as an indicator of acute inflammation in the clinic.

## Data Availability Statement

The original contributions presented in the study are included in the article/**Supplementary Material**. Further inquiries can be directed to the corresponding authors.

## Ethics Statement

Ethical review and approval was not required for the study on human participants in accordance with the local legislation and institutional requirements. Written informed consent from the participants’ legal guardian/next of kin was not required to participate in this study in accordance with the national legislation and the institutional requirements.

## Author Contributions

AJS conceived the study design, performed literature analysis, performed transcriptomic analyses, and wrote the manuscript. MG assisted with analysis conceptualization. DC and CF supervised the work. All authors contributed to the article and approved the submitted version.

## Funding

AJS is supported by an Australian Government Research Training Program (RTP) Scholarship.

## Conflict of Interest

The authors declare that the research was conducted in the absence of any commercial or financial relationships that could be constructed as a potential conflict of interest.

## Supplementary Material

Supplementary Table 1 Characteristics of the public datasets used in this study.
