# Supplementary material for: Transcriptomic Profiling Identifies Neutrophil-Specific Upregulation of Cystatin F as a Marker of Acute Inflammation in Humans
Source: Front Immunol. 2021 Apr 1;12:634119. doi: 10.3389/fimmu.2021.634119 (PMC8047108; doi:10.3389/fimmu.2021.634119)
Supplement: Supplementary Table 1 — Characteristics of the public datasets used in this study. [file Table_1.docx]

|  | Date | References (PMID) | Samples used | Species | Age | Cell type | Study type | Assay type | Groups used in our study |
| --- | --- | --- | --- | --- | --- | --- | --- | --- | --- |
| **GSE2322** | 01/01/06 | 16861384 | 58 | Homo sapiens | Adult | Airspace  neutrophils  Circulating neutrophils | *In vivo*  *In vitro* | Affymetrix  Microarray | Airspace neutrophils  *In vitro* control  *In vitro* endotoxin  *In vivo* control  *In vivo* endotoxin |
| **GSE8668** | 19/11/07 | 18006867 | 24 | Homo sapiens | 12–29 | Purified  neutrophils | *In vivo* | Affymetrix  Microarray | Pre-exercise  Post-exercise |
| **GSE13015** | 01/07/09 | 19903332 | 106 | Homo sapiens | 37–74 | Whole blood | *In vivo* | Illumina  Microarray | Healthy control  Sepsis |
| **GSE17755** | 21/08/10 | 21496236 | 53 | Homo sapiens | 2–19 | Whole blood | *In vivo* | Affymetrix  Microarray | Healthy child  SOJIA |
| **GSE19491** | 11/08/10 | 20725040 | 80 | Homo sapiens | 21–72 | Whole blood | *In vivo* | Illumina  Microarray | Healthy control  Tuberculosis |
| **GSE25504** | 12/08/14 | 26484146 | 61 | Homo sapiens | Neonate | Whole blood | *In vivo* | Illumina  Microarray | Healthy child  Sepsis |
| **GSE25742** | 26/10/14 | 25344726 | 22 | Homo sapiens | 2–42 | Whole blood | *In vivo* | Illumina  Microarray | Healthy control  MYD88 deficiency  IRAK4 deficiency |
| **GSE27131** | 01/04/11 | 21781987 | 14 | Homo sapiens | 28–59 | Whole blood | *In vivo* | Affymetrix  Microarray | Healthy control  H1N1 |
| **GSE28623** | 28/10/11 | 25895988 | 83 | Homo sapiens | Adult | Whole blood | *In vivo* | Agilent  Microarray | Healthy control  Active tuberculosis |
| **GSE28750** | 01/11/11 | 21682927 | 30 | Homo sapiens | Adult | Whole blood | *In vivo* | Affymetrix  Microarray | Healthy control  Sepsis |
| **GSE29366** | 12/05/15 | - | 31 | Homo sapiens | 0–3 | Whole blood | *In vivo* | Illumina  Microarray | Healthy child  Influenza |
| **GSE29536** | 11/03/14 | 22496797 | 170 | Homo sapiens | Adult & children | Whole blood | *In vivo* | Illumina Microarray | Tuberculosis control  Tuberculosis  Influenza control  Influenza  SOJIA control  SOJIA  Sepsis control  Sepsis |
| **GSE30101** | 20/06/11 | 23601689 | 72 | Homo sapiens | 22–41 | Whole blood | *In vitro* | Illumina Microarray | Unstimulated blood  Stimulated blood |
| **GSE30119** | 26/06/11 | 22496797 | 143 | Homo sapiens | 0–18 | Whole blood | *In vivo* | Illumina Microarray | Healthy child  *S. Aureus* infection |
| **GSE34608** | 21/12/11 | 22547807 | 26 | Homo sapiens | Adult | Whole blood | *In vivo* | Agilent  Microarray | Healthy control  Tuberculosis |
| **GSE38900** | 12/11/13 | 24265599 | 154 | Homo sapiens | 0–2 | Whole blood | *In vivo* | Illumina  Microarray | Healthy Control  RSV  IAV |
| **GSE40012** | 15/08/12 | 22898401 | 24 | Homo sapiens | Adult | Whole blood | *In vivo* | Illumina  Microarray | Healthy Control  IAV pneumonia |
| **GSE41055** | 05/02/13 | 23375113 | 18 | Homo sapiens | 1–14 | Whole blood | *In vivo* | Affymetrix  Microarray | Healthy control  Tuberculosis |
| **GSE42026** | 05/09/13 | 23901082 | 74 | Homo sapiens | Adult | Whole blood | *In vivo* | Illumina  Microarray | Healthy control  H7N9  RSV |
| **GSE42834** | 31/10/13 | 23940611 | 148 | Homo sapiens | Adult | Whole blood | *In vivo* | Illumina  microarray | Healthy control  Tuberculosis |
| **GSE49757** | 11/07/16 | 24612859 | 46 | Homo sapiens | Adult | Purified neutrophils | *In vitro* | Illumina  Microarray | Stimulation with healthy plasma  Stimulation with septic plasma |
| **GSE54514** | 04/02/14 | 23807251 | 53 | Homo sapiens | 24–86 | Whole blood | *In vivo* | Illumina Microarray | Healthy  Sepsis survivor  Sepsis non-survivor |
| **GSE57183** | 03/09/15 | 26267155 | 10 | Homo sapiens | 1–11 | Whole blood | *In vivo* | Illumina  Microarray | Healthy control  SOJIA Kawasaki disease |
| **GSE60424** | 06/01/15 | 25314013 | 14 | Homo sapiens | Adult | Purified neutrophils,  Whole blood | *In vivo* | Illumina RNAseq | Healthy control  Sepsis |
| **GSE64457** | 28/12/15 | 26224052 | 23 | Homo sapiens | Adult | Purified Neutrophils | *In vivo* | Affymetrix  Microarray | Healthy control  Septic shock patients (w/ sepsis induced immunosuppression) |
| **GSE66099** | 12/05/15 | 27635771 | 65 | Homo sapiens | Adult | Whole blood | *In vivo* | Affymetrix  Microarray | Healthy control  Sepsis |
| **GSE69528** | 04/06/15 | 19903332 | 72 | Homo sapiens | Adult | Whole blood | *In vivo* | Illumina  Microarray | Healthy control  Sepsis |
| **GSE71010** | 18/09/15 | 27271962 | 93 | Homo sapiens | Children | Purified neutrophils | *In vivo* | Affymetrix  Microarray | Healthy  Cystic fibrosis  SOJIA |
| **GSE76293** | 11/04/16 | 27064380 | 22 | Homo sapiens | Adult | Purified neutrophils | *In vivo* | Affymetrix  Microarray | Healthy control  ARDS |
| **GSE80060** | 29/04/16 | 28115015 | 134 | Homo sapiens | Children | Whole blood | *In vivo* | Affymetrix  Microarray | Healthy control  SOJIA prior to treatment |
| **GSE81746** | 24/05/16 | - | 7 | Homo sapiens | 22–65 | Whole blood | *In vivo* | Agilent  Microarray | Healthy control  Tuberculosis |
| **GSE83456** | 04/11/16 | 27706152 | 106 | Homo sapiens | 19–82 | Whole blood | *In vivo* | Illumina  Microarray | Healthy control  Pulmonary tuberculosis |
| **GSE100151** | 16/06/20 | - | 63 | Homo sapiens | Adult | Whole blood | *In vivo* | Illumina  Microarray | Healthy control  HIV |
| **GSE100154** | 16/06/20 | - | 44 | Homo sapiens | Children | Whole blood | *In vivo* | Illumina  Microarray | Healthy control  Kawasaki disease |
| **GSE100159** | 16/06/20 | - | 27 | Homo sapiens | Adult | Whole blood | *In vivo* | Illumina  Microarray | Healthy control  Sepsis |
| **GSE100160** | 16/06/20 | - | 39 | Homo sapiens | Adult | Whole blood | *In vivo* | Illumina  Microarray | Healthy control  Flu |
| **GSE100161** | 16/06/20 | - | 84 | Homo sapiens | Adult | Whole blood | *In vivo* | Illumina  Microarray | Healthy control  RSV |
| **GSE100164** | 16/06/20 | - | 85 | Homo sapiens | Children | Whole blood | *In vivo* | Illumina  Microarray | Healthy Control  SOJIA |
| **GSE100166** | 16/06/20 | - | 34 | Homo sapiens | Adult | Whole blood | *In vivo* | Illumina  Microarray | Healthy control  Tuberculosis |
| **GSE101584** | 19/07/17 | 27999744 | 15 | Mus musculus | - | Purified neutrophils | *In vitro* | Illumina Microarray | Healthy neutrophils  Tumour associated neutrophils  TAN + SM16 |
| **GSE103170** | 12/02/18 | 29426067 | 6 | Homo sapiens | Children | Purified  neutrophils | *In vivo* | Illumina  RNAseq | Healthy control  SOJIA |
| **GSE107731** | 06/12/17 | - | 6 | Homo sapiens | Adult | Whole  blood | In vivo | Affymetrix  Microarray | Healthy control  Tuberculosis |
| **GSE111368** | 03/03/18 | 29777224 | 329 | Homo sapiens | 18–71 | Whole  blood | In vivo | Illumina Microarray | Healthy control  H1N1 |
